# Short-Term and Long-Term Risk of Recurrent Vascular Event by Cause After Ischemic Stroke in Young Adults

**DOI:** 10.1001/jamanetworkopen.2024.0054

**Published:** 2024-02-20

**Authors:** Esmée Verburgt, Nina A. Hilkens, Merel S. Ekker, Mijntje M. I. Schellekens, Esther M. Boot, Maikel H. M. Immens, Mayte E. van Alebeek, Paul J. A. M. Brouwers, Renate M. Arntz, Gert W. van Dijk, Rob A. R. Gons, Inge W. M. van Uden, Tom den Heijer, Julia H. van Tuijl, Karlijn F. de Laat, Anouk G. W. van Norden, Sarah E. Vermeer, Marian S. G. van Zagten, Robert J. van Oostenbrugge, Marieke J. H. Wermer, Paul J. Nederkoorn, Henk Kerkhoff, Fergus A. Rooyer, Frank G. van Rooij, Ido R. van den Wijngaard, Tim J. F. ten Cate, Anil M. Tuladhar, Frank-Erik de Leeuw, Jamie I. Verhoeven

**Affiliations:** 1 Donders Institute for Brain, Cognition, and Behaviour, Department of Neurology, Radboud University Medical Centre, Nijmegen, the Netherlands; 2 Department of Neurology, Gelre Hospital, Apeldoorn, the Netherlands; 3 Department of Neurology, Medisch Spectrum Twente, Enschede, the Netherlands; 4 Department of Neurology, Canisius-Wilhelmina Hospital, Nijmegen, the Netherlands; 5 Department of Neurology, Catharina Hospital, Eindhoven, the Netherlands; 6 Department of Neurology, Sint Franciscus Gasthuis Hospital, Rotterdam, the Netherlands; 7 Department of Neurology, Elisabeth-TweeSteden Hospital, Tilburg, the Netherlands; 8 Department of Neurology, Haga Hospital, The Hague, the Netherlands; 9 Department of Neurology, Amphia Hospital, Breda, the Netherlands; 10 Department of Neurology, Rijnstate Hospital, Arnhem, the Netherlands; 11 Department of Neurology, Jeroen Bosch Hospital, ‘s-Hertogenbosch, the Netherlands; 12 Department of Neurology, Maastricht University Medical Centre, Maastricht, the Netherlands; 13 Department of Neurology, Leiden University Medical Centre, Leiden, the Netherlands; 14 Department of Neurology, University Medical Centre Groningen, Groningen, the Netherlands; 15 Department of Neurology, Amsterdam University Medical Centre, Amsterdam, the Netherlands; 16 Department Neurology, Albert Schweitzer Hospital, Dordrecht, the Netherlands; 17 Department of Neurology, Zuyderland Hospital, Sittard-Geleen, the Netherlands; 18 Medical Centre Leeuwarden, Department of Neurology, Leeuwarden, the Netherlands; 19 Department of Neurology, Haaglanden Medical Centre, The Hague, the Netherlands; 20 Department of Cardiology, Radboud University Medical Centre, Nijmegen, the Netherlands

## Abstract

**Importance:**

Cause of ischemic stroke in young people is highly variable; however, the risk of recurrence is often presented with all subtypes of stroke grouped together in classification systems such as the Trial of ORG (danaparoid sodium [Orgaran]) 10172 in Acute Stroke Treatment (TOAST) criteria, which limits the ability to individually inform young patients with stroke about their risk of recurrence.

**Objective:**

To determine the short-term and long-term risk of recurrent vascular events after ischemic stroke at a young age by stroke cause and to identify factors associated with recurrence.

**Design, Setting, and Participants:**

This cohort study used data from the Observational Dutch Young Symptomatic Stroke Study, a prospective, multicenter, hospital-based cohort study, conducted at 17 hospitals in the Netherlands between 2013 and 2021. Eligible participants included 30-day survivors of an initial, neuroimaging-proven ischemic stroke (aged 18-49 years). Data analysis was conducted from June to July 2023.

**Exposure:**

Diagnosis of a first-ever, ischemic stroke via neuroimaging.

**Main Outcome and Measures:**

The primary outcome was short-term (within 6 months) and long-term (within 5 years) recurrence risk of any vascular event, defined as fatal or nonfatal recurrent ischemic stroke, transient ischemic attack, myocardial infarction, and revascularization procedure. Predefined characteristics were chosen to identify factors associated with risk of recurrence (cause of stroke, age, sex, stroke severity, and cardiovascular health factors).

**Results:**

A total of 1216 patients (median [IQR] age, 44.2 [38.4-47.7] years; 632 male [52.0%]; 584 female [48.0%]) were included, with a median (IQR) follow-up of 4.3 (2.6-6.0) years. The 6-month risk of any recurrent ischemic event was 6.7% (95% CI, 5.3%-8.1%), and the 5-year risk was 12.2% (95% CI, 10.2%-14.2%)The short-term risk was highest for patients with cervical artery dissections (13.2%; 95% CI, 7.6%-18.7%). Other factors associated with a recurrent short-term event were atherothrombotic stroke, rare causes of stroke, and hypertension. The long-term cumulative risk was highest for patients with atherothrombotic stroke (22.7%; 95% CI, 10.6%-34.7%) and lowest for patients with cryptogenic stroke (5.8%; 95% CI, 3.0%-8.5%). Cardioembolic stroke was associated with a recurrent long-term event, as were diabetes and alcohol abuse.

**Conclusions and Relevance:**

The findings of this cohort study of 1216 patients with an ischemic stroke at a young age suggest that the risk of recurrent vascular events was high and varied by cause of stroke both for short-term and long-term follow-up, including causes that remained concealed when combined into 1 category in the routinely used TOAST criteria. This knowledge will allow for more personalized counseling of young patients with stroke.

## Key Points

**Question:**

What is the short-term and long-term risk of recurrent vascular events by stroke cause after ischemic stroke at a young age?

**Findings:**

In this cohort study of 1216 patients with ischemic stroke at a young age, the 5-year risk of any recurrent vascular event was 12.2%; this risk was highest for patients with atherothrombotic stroke and lowest for patients with cryptogenic stroke. Patients with stroke due to cervical artery dissection had the highest risk of short-term recurrence (within 6 months) after stroke.

**Meaning:**

These findings suggest that the risk of recurrent vascular events after stroke at a young age is high and differs according to time interval and cause of stroke.

## Teaser

This cohort study assesses the risk of recurrent vascular events for young adults with ischemic stroke in the Netherlands.

## Introduction

The incidence of ischemic stroke in young adults has almost doubled within the last 2 decades, with an attendant increase in the medical and socioeconomic burden.^1,2,3,4^ Stroke at a young age has a major and long-lasting impact because patients still have a long life expectancy and are in a life phase in which they have yet to reach important milestones in their personal lives and careers. An important concern after a first-ever stroke is the risk of a recurrent vascular event, which increases the risk of mortality and disability and reduces quality of life in these young patients.^5,6,7^

Stroke in the young is a much more heterogeneous disease compared with the older population. As a consequence, findings on the recurrence risk in older patients cannot simply be extrapolated to younger patients. Despite the many varying causes of stroke in young adults, the risk of recurrence is often presented years after the event and groups all subtypes of stroke together.^8,9,10^ Studies investigating the short-term and long-term risk of recurrent vascular events per cause of stroke in young patients are scarce, which limits the ability to individually inform young patients about their risk of recurrence. Previous studies^8,9,10,11,12^ had small sample sizes, were conducted before the implementation of current secondary prevention regimes, and/or included patients without neuroimaging proof of stroke. Inclusion of patients without neuroimaging proof of stroke has led to an underestimation of the risk of recurrence because stroke mimics have a low risk of future vascular events.^13^

The aim of this study was to investigate the short-term and long-term risk of recurrent vascular events by cause in the current era of routinely used secondary prevention and to identify subgroups of patients at high-risk of recurrence after a first-ever, neuroimaging-proven ischemic stroke at a young age.

## Methods

### Study Population and Study Design

This cohort study was approved by the Oost-Nederland medical ethical review board and all patients provided written informed consent. The study followed the Strengthening the Reporting of Observational Studies in Epidemiology (STROBE) reporting guideline for cohort studies. Patients were included from the multicenter prospective Observational Dutch Young Symptomatic StrokE studY (ODYSSEY).^14^ The ODYSSEY included consecutive patients with first-ever stroke aged 18 and 49 years who were recruited from the neurology departments of 17 hospitals in the Netherlands. Patients were included between May 2013 and February 2021. For this study, patients with an ischemic stroke were included; ischemic stroke was defined as an acute neurological deficit with a corresponding lesion seen on a computed tomography, computed tomography angiography, magnetic resonance imaging, or magnetic resonance angiography scan. Because patients with transient symptoms (<24 hours) were also required to have imaging proof of ischemia, they were classified as minor strokes according to the tissue based definition.^15^ Exclusion criteria were a history of transient ischemic attack (TIA; including transient monocular blindness or retinal infarction) or ischemic stroke, intracerebral hemorrhage (ICH), and cerebral venous sinus thrombosis. For this study, we excluded patients who died within 30 days of their index event.

### Risk Factors

Age was divided into categories (18-29 years, 30-34 years, 35-39 years, 40-44 years, and 45-49 years). The following cardiovascular factors associated with increased risk of stroke were systematically assessed through medical files and diagnostic tests at time of index event: hypertension (known history of hypertension, use of antihypertensive medication, a systolic blood pressure >140 mm Hg, and/or a diastolic blood pressure >90 mm Hg at least 24 hours after index event), dyslipidemia (known history of dyslipidemia, use of statins, total cholesterol >193.05 mg/dL, low-density lipoprotein cholesterol >115.83 mg/dL, or high density lipoprotein cholesterol <38.61 mg/dL [to convert cholesterol to millimoles per liter, multiply by .0259]), diabetes (known history of diabetes, use of diabetic medication, 2 values of glucose >126.13 mg/dL [to convert to millimoles per liter, multiply by .0555], or 1 value of hemoglobin A_1C _ >6.5% [to change to proportion of total hemoglobin, multiply by .01]), smoking (currently smoking, previous smoking, or never smoked), excessive alcohol use (>200 g of alcohol per week), drug use (use of cocaine, heroin, methadone, amphetamines, cannabis, or ecstasy at least once a week in the year before the stroke), obesity (a body mass index ≥30 [body mass index was calculated as weight in kilograms divided by height in meters squared]), and the use of oral contraceptives.

### Stroke Characteristics

Cause of index stroke was based on the modified Trial of ORG (danaparoid sodium [Orgaran]) 10172 in Acute Stroke Treatment (TOAST) criteria^16^ with the additional subcategory, cervical artery dissection (CeAD), which is defined in the eAppendix in Supplement 1. eTable 1 in Supplement 1 shows the diagnostic testing in patients with cryptogenic stroke. Stroke severity at admission was assessed with the National Institute of Health Stroke Scale (NIHSS).^17^ A NIHSS score of 3 or less was defined as a minor stroke and a score greater than 3 was defined as a major stroke.^18^ The use of antithrombotic therapy at discharge was systematically assessed.

### Follow-Up

Recurrent events were collected through standardized, structured questionnaires by phone between November 2013 and June 2022. Patients or their primary caregiver were asked about the occurrence of any fatal or nonfatal vascular event, including stroke, TIA, myocardial infarction, coronary artery bypass grafting, percutaneous transluminal coronary angioplasty, or other revascularization procedure. If an event was reported, medical records were retrieved from the including hospital. If the hospital did not have medical records matching the event, we reached out to the general practitioner. Accuracy of this patient-reported approach was verified by cross-validating the medical records of 64 patients who reported no events from 6 of 17 hospitals, which resulted in 100% agreement. The events and use of antithrombotic treatment were classified by trained raters and adjudicated by experts of the corresponding fields.

### Recurrent Vascular Event

Recurrent vascular ischemic events were defined as ischemic stroke or TIA, myocardial infarction, revascularization procedures, or vascular death. Recurrent ischemic stroke was defined as an acute neurological deficit (with only a vascular explanation) lasting longer than 24 hours. TIA was defined similarly, but with symptoms lasting less than 24 hours.^19^ Neurological deterioration within 24 hours of the index event was not classified as a recurrent stroke. Myocardial infarction was defined as symptomatic, acute coronary syndrome including changes on electrocardiogram and/or elevated cardiac troponin values, with or without intervention. Other arterial revascularization procedures not due to the index stroke were also classified as an event. Vascular death was defined as death within 30 days after any of the previously mentioned recurrent events. ICH was not classified as a recurrent event given the different pathophysiological mechanisms compared with ischemic events. Short-term recurrent events were defined as any of these events occurring within 6 months after the index event, and long-term recurrent events were defined as any of these events occurring within 5 years.

### Statistical Analyses

Differences in baseline characteristics between patients with and without a recurrent event were examined with an independent *t* test, Mann-Whitney U test, or χ^2^ test, where appropriate. The number of patient-years was calculated from index stroke to date of either first recurrent vascular event, last available follow-up, or death. The cumulative incidence was assessed by type of recurrent vascular event (ischemic stroke and TIA or other vascular events) and the modified TOAST classification with the cumulative incidence function, accounting for competing risk of death. We performed a sensitivity analysis to calculate the 5-year cumulative incidence for all types of recurrent vascular events, excluding recurrent TIAs. TIAs have a relatively high risk of misclassification, and, thus, may provide an overestimation of the risk.^20^ The incidence rate per 100 person-years for each type of recurrent event was calculated by dividing the number of events by the total person-years at risk per 6-month intervals up to 5 years; for cause of stroke, this was calculated in intervals including the first 6 months, 6 months to 1 year, 1 to 2 years, and 2 to 5 years.

Age- and sex-corrected Fine-Gray proportional hazard models with competing risk of death were used to determine the hazard ratio (HR) of the short-term (<6 months) and long-term (6 months to 5 years) risk of any vascular event per predefined factors (modified TOAST classification, age, sex, stroke severity, and cardiovascular risk factors). Multivariable analyses were performed including factors with a *P* value < .10 in the age- and sex-corrected analyses. The assumption of proportional hazards was tested through analysis of the Schoenfeld residuals and were met. Significance level was predefined as *P* < .05. All tests were 2-sided and were performed between June and July 2023 with R statistical software version 3.6.0 (R Project for Statistical Computing).

## Results

**Figure 1.**
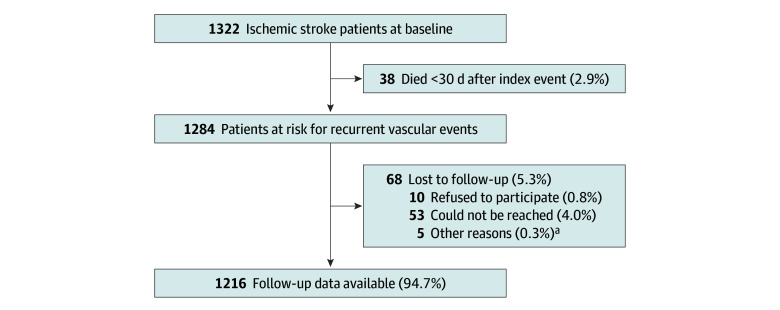
Patient Inclusion ^a^Other reasons for loss to follow-up were patients with aphasia, not speaking the Dutch language, or patients being too affected by the stroke to participate during follow-up.

In total, 1216 patients (median [IQR] age, 44.2 [38.4-47.7] years; 584 female [48.0%]; 632 male [52.0%]) were included in this study (Figure 1). Table 1 shows the characteristics of the patients and eTable 2 in Supplement 1 shows the distribution of causes in patients with multiple causes. The most common TOAST classification was cryptogenic stroke (302 patients [24.8%]), which was the case for all age groups. Of the 52 patients with atherothrombotic stroke, 40 (76.9%) occurred in patients aged 45 to 49 years (eTable 3 in Supplement 1). Female participants more often had a rare cause of stroke or cryptogenic stroke and less often an atherothrombotic stroke or small vessel disease stroke compared with male participants. eTable 4 in Supplement 1 shows the use of antithrombotic treatment at discharge and recurrence per cause of stroke. Median (IQR) follow-up was 4.3 (2.6-6.0) years, with a total of 4743 patient-years.

**Table 1.** Patient Characteristics

Characteristic	Patients, No. (%)			*P* value
	All (N =1216)	Without recurrent event during follow-up (n = 1079)	With recurrent event during follow-up (n = 137)	
Age, y				
Median (IQR)	44.2 (38.4-47.7)	44.1 (37.8-47.3)	45.5 (40.6-48.3)	.006
18-29	124 (100)	116 (93.5)	8 (6.5)	.09
30-34	92 (100)	86 (93.5)	6 (6.5)	
35-39	154 (100)	139 (90.3)	15 (9.7)	
40-44	296 (100)	262 (88.5)	34 (11.5)	
45-49	550 (100)	476 (86.5)	74 (13.5)	
Sex				
Male	632 (52.0)	554 (51.3)	78 (56.9)	.25
Female	584 (48.0)	525 (48.7)	59 (43.1)	
Follow-up, median (IQR), y	4.3 (2.6-6.0)	4.3 (2.4-5.9)	5.2 (3.1-6.8)	.001
Stroke characteristics				
TOAST stroke classification				
Atherothrombotic	52 (4.3)	41 (3.8)	11 (8.0)	.008
Likely atherothrombotic	166 (13.7)	147 (13.6)	19 (13.9)	
Small vessel disease	165 (13.6)	142 (13.2)	23 (16.8)	
Cardioembolic	200 (16.4)	180 (16.7)	20 (14.6)	
Rare cause	130 (10.7)	112 (10.4)	18 (13.1)	
Cervical artery dissection	144 (11.8)	123 (11.4)	21 (15.3)	
Cryptogenic	302 (24.8)	285 (26.4)	17 (12.4)	
Multiple causes	57 (4.7)	49 (4.5)	8 (5.8)	
National Institute of Health Stroke Scale score at admission, median (IQR)^a^	3 (1-6)	3 (1-6)	3 (1-5)	
≤3 (minor stroke)	726 (59.7)	644 (59.7)	82 (59.9)	.54
>3 (major stroke)	486 (40.0)	431 (39.9)	55 (40.1)	
Vascular health factors				
Hypertension	459 (37.7)	387 (35.9)	72 (52.6)	<.001
Dyslipidemia	801 (65.9)	708 (65.6)	93 (67.9)	.67
Diabetes	118 (9.7)	92 (8.5)	25 (18.2)	<.001
Smoking				
Current	267 (22.0)	228 (21.1)	39 (28.5)	.13
Previous^b^	459 (37.7)	405 (37.9)	54 (39.4)	
Excess alcohol use	81 (6.7)	67 (6.2)	14 (10.2)	.11
Illicit drug use^c^	101 (10.2)	89 (8.2)	12 (8.8)	.59
Obesity	253 (20.8)	222 (20.6)	31 (22.6)	.66
Use of oral contraceptives^d^	277 (47.4)	251 (47.8)	26 (44.1)	.27
No. of vascular health factors^e^				
0	136 (11.2)	123 (11.4)	13 (9.5)	<.001
1-2	661 (54.4)	600 (55.6)	61 (44.5)	
≥3	419 (34.5)	356 (33.0)	63 (46.0)	

Abbreviation: TOAST, Trial of ORG (danaparoid sodium [Orgaran]) 10172 in Acute Stroke Treatment.

^a^ The National Institute of Health Stroke Scale was not available for 4 patients.

^b^ A total of 115 patients reported ever having smoked, but it is not known whether they were smoking at time of event.

^c^ Information about illicit drug use was not available for 226 patients.

^d^ Percentage of oral contraceptives use was calculated for female participants only.

^e^ No. of vascular risk factors was determined by the presence of hypertension, dyslipidemia, diabetes, smoking, excess alcohol use, and obesity.

There were 170 recurrent vascular events identified in 137 patients (11.3%) during follow-up. There were 82 patients (6.7%) who had their first recurrence within 6 months. A total of 28 patients had more than 1 recurrent vascular event, of whom 11 had both short-term and long-term recurrences. The median (IQR) interval until the first recurrent event was 3.3 months (1 week to 35 months). Of the 170 recurrent events, 93 (54.7%) were ischemic strokes, 51 (30.0%) were TIAs, 17 (10.0%) were myocardial infarctions (of which 2 were fatal) and 9 (5.3%) were revascularization procedures. In addition to the 2 deaths from myocardial infarction, 33 other patients (2.9%) died during follow-up; 16 died from a nonvascular cause and for 17 patients the cause of death could not be determined because the treating hospitals did not have the corresponding medical documents. During follow-up, 4 intracranial bleeds were identified; none were spontaneous ICHs. A total of 108 patients with a recurrent event (88.5%) were using antithrombotic treatment at the time of the event. Twelve patients (9.8%) had a recurrent event shortly after the index event and had not started therapy yet.

### Risk of Recurrent Vascular Events

**Figure 2.**
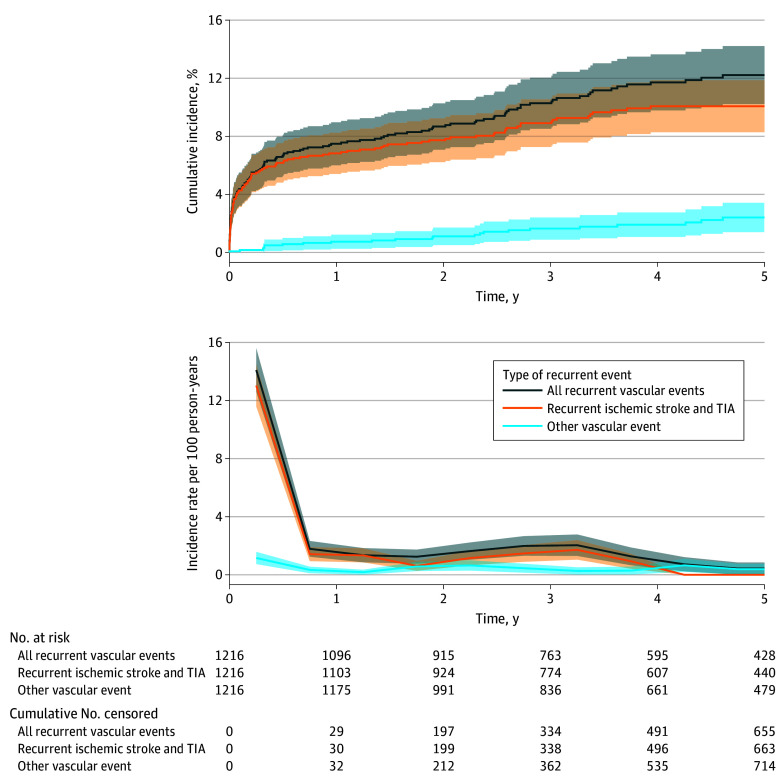
Five-Year Cumulative Incidence and Incidence Rate Per 100 Person-Years by Type of Recurrent Event The cumulative incidence was calculated through cumulative incidence functions. The incidence rate per 100 person-years was calculated by dividing the number of events by the total person-years at risk per 6-month intervals. The middle of those intervals was chosen as the point estimator in the graph. Shaded regions indicate 95% CIs. TIA indicates transient ischemic attack.

The cumulative 6-month incidence was 6.7% (95% CI, 5.3%-8.1%) for any vascular event, 6.2% (95% CI, 4.8%-7.5%) for recurrent ischemic stroke or TIA, and 0.6% (95% CI, 0.2%-1.0%) for other vascular events. The cumulative 5-year incidence was 12.2% (95% CI, 10.2%-14.2%) for any vascular event, 10.1% (95% CI, 8.3%-11.9%) for recurrent ischemic stroke or TIA, and 2.4% (95% CI, 1.4%-3.4%) for any other vascular event. The cumulative 5-year incidence after excluding TIAs as outcome event was 9.5% (95% CI, 7.7%-11.3%). The incidence rate per 100 person-years for any vascular event (14.1; 95% CI, 12.6-15.6) and recurrent stroke or TIA (13.0; 95% CI, 11.6-14.5) was highest in the first 6 months after stroke; between 6 months and 1 year, the incidence rate per 100 person-years decreased to 1.8 (95% CI, 1.2-2.3) for any vascular event and 1.4 (95 CI, 0.9-1.9) for recurrent stroke or TIA. For other recurrent events, the incidence rate per 100 person-years was 1.2 (95% CI, 0.7-1.6) in the first 6 months and 0.3 (95% CI, 0.1-0.6) between 6 months and 1 year (Figure 2).

### Risk of Recurrent Vascular Event by Cause of Stroke

**Figure 3.**
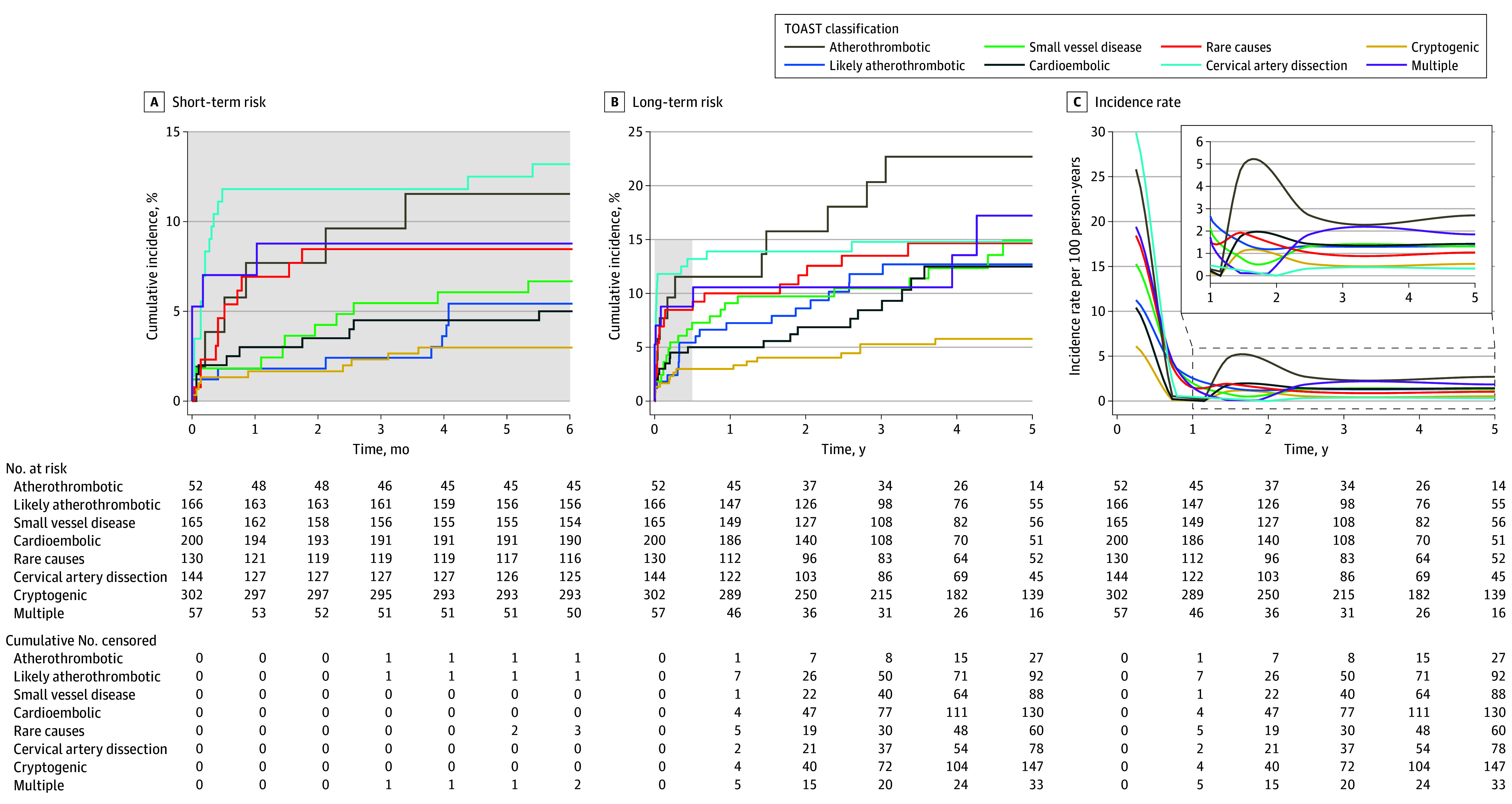
Cumulative Incidence and Incidence Rate Per 100 Person-Years Per Cause of Stroke Cumulative incidence is shown for (A) short-term recurrence (<6 months) and (B) long-term recurrence (5 years). Recurrence was calculated through cumulative incidence functions. The incidence rate per 100 person-years (C) was calculated by dividing the number of events by the total person-years at risk in the first 6 months, 6 months to 2 years, and 2 to 5 years. The middle of those intervals was chosen as the point estimator in the graph. Shaded region indicates the short-term period. TOAST indicates Trial of ORG (danaparoid sodium [Orgaran]) 10172 in Acute Stroke Treatment.

The cumulative risk of any recurrent event for atherothrombotic stroke was 11.5% (95% CI, 2.8%-20.3%) in the short term and 22.7% (95% CI, 10.6%-34.7%) in the long term. The short-term cumulative risk was 5.4% (95% CI, 2.0%-8.9%) for likely atherothrombotic stroke, 7.3% (95% CI, 3.3%-11.2%) for small vessel disease, 5.0% (95% CI, 2.0%-8.0%) for cardioembolic stroke, 8.5% (95% CI, 3.7%-13.3%) for rare causes of stroke, 13.2% (95% CI, 7.6%-18.7%) for CeAD, 8.8% (95% CI, 1.4%-16.2%) for multiple-cause stroke, and 3.0% (95% CI, 1.1%-4.9%) for cryptogenic stroke. The long-term cumulative incidence was 12.7% (95% CI, 7.3%-18.1%) for likely atherothrombotic stroke, 14.9% (95% CI, 8.7%-21.2%) for small vessel disease, 12.5% (95% CI, 6.9%-18.1%), for cardioembolic stroke, 14.7% (95% CI, 8.3%-21.0%) for rare causes of stroke, 14.8% (95% CI, 8.9%-20.7%) for CeAD, 17.2% (95% CI, 5.4%-29.0%) for multiple-cause stroke, and 5.8% (95% CI, 3.0%-8.5%) for cryptogenic stroke. The incidence rate per 100 person-years after an atherothrombotic stroke decreased from 23.2 (95% CI, 15.7-35.9) in the first 6 months to 2.7 (95% CI, 1.2-4.2) between 2 and 5 years. The rate per 100 person-years decreased from 11.2 (95% CI, 7.6-14.8) to 1.3 (95% CI, 0.8-1.9) for likely atherothrombotic stroke, from 15.3 (95% CI, 11.0-19.5) to 1.3 (95% CI, 0.7-1.9) for small vessel disease, from 10.4 (95% CI, 7.2-13.6) to 1.4 (95% CI, 0.9-2.2%) for cardioembolic stroke, from 18.4 (95% CI, 13.1-23.8) to 1.0 (95% CI, 0.5-1.6) for rare causes of stroke, from 29.9 (95% CI, 23.3-36.5) to 0.3 (95% CI, 0.0-0.6) for CeAD, from 6.1 (95% CI, 4.1-8.1) to 0.5% (95% CI, 0.3-0.8) for cryptogenic stroke, and from 19.4 (95% CI, 11.1-27.7) to 1.9 (95% CI, 0.6-3.1) for multiple-cause stroke (Figure 3).

### Factors Associated With Any Recurrent Event

The age- and sex-adjusted HRs and multivariable HRs per predefined characteristic are presented in Table 2. The risk of any short-term vascular event in the multivariable analysis was significantly higher for patients with CeAD (HR, 4.6; 95% CI, 2.1-10.2; *P* < .001), atherothrombotic stroke (HR, 3.5; 95% CI, 1.3-9.5; *P* = .02), and other rare cause of stroke (HR, 3.2; 95% CI, 1.3-9.5; *P* = .008) compared with cryptogenic stroke. Hypertension was also associated with an increased risk of any short-term vascular event (HR, 1.9; 95% CI, 1.2-2.9; *P* = .006). The long-term risk was significantly higher for patients with cardioembolic stroke compared with cryptogenic stroke (HR, 2.6; 95% CI, 1.1-6.5; *P* = .04). There was also an increased long-term risk for patients with a history of diabetes (HR, 2.9; 95% CI, 1.7-4.9; *P* < .001) and alcohol abuse (HR, 2.0; 95% CI, 1.0-3.8; *P* = .04).

**Table 2.** Factors Associated With Any Short-Term and Long-Term Recurrent Vascular Events

Factor	Short-term recurrent event (<6 mo after index stroke)				Long-term recurrent event (>6 mo to 5 y)			
	Age- and sex-adjusted, HR (95% CI)^a^	*P* value	Multivariable, HR (95% CI)^b^	*P* value	Age- and sex-adjusted, HR (95% CI)^a^	*P* value	Multivariable, HR (95% CI)^b^	*P* value
Sex								
Female	0.8 (0.5-1.3)	.36	NA	NA	0.8 (0.5-1.3)	.37	NA	NA
Male	1 [Reference]	NA	NA	NA	1 [Reference]	NA	NA	NA
Age, y								
18-29	0.8 (0.4-1.8)	.57	NA	NA	0.1 (0.0-0.8)	.03	0.2 (0.0-1.3)	.09
30-34	0.6 (0.2-1.7)	.33	NA	NA	0.4 (0.1-1.4)	.18	0.6 (0.2-2.1)	.45
35-39	0.9 (0.5-1.8)	.76	NA	NA	0.4 (0.2-1.1)	.08	0.5 (0.2-1.3)	.16
40-44	1.0 (0.6-1.7)	.95	NA	NA	0.7 (0.4-1.2)	.18	0.8 (0.4-1.4)	.36
45-49	1 [Reference]	NA	NA	NA	1 [Reference]	NA	1 [Reference]	NA
Stroke cause								
Atherothrombotic	3.7 (1.3-10.6)	.01	3.5 (1.3-9.5)	.02	3.4 (1.2-9.6)	.02	2.0 (0.7-6.0)	.21
Likely atherothrombotic	1.7 (0.7-4.5)	.24	1.5 (0.6-3.7)	.43	2.6 (1.0-6.2)	.04	1.6 (0.7-4.0)	.30
Small vessel disease	2.3 (1.0-5.6)	.06	1.8 (0.8-4.3)	.19	2.4 (1.0-5.7)	.06	1.5 (0.6-4.0)	.42
Cardioembolic source	1.7 (0.7-4.1)	.25	1.6 (0.7-4.0)	.28	2.9 (1.2-7.2)	.02	2.6 (1.1-6.5)	.04
Rare cause	3.3 (1.4-7.8)	.008	3.2 (1.3-9.5)	.008	2.3 (0.8-6.4)	.11	2.2 (0.8-6.1)	.14
Cervical artery dissection	4.6 (2.1-10.3)	<.001	4.6 (2.1-10.2)	<.001	1.3 (0.4-4.1)	.61	1.2 (0.4-3.7)	.69
Multiple	3.0 (1.0-9.1)	.05	2.3 (0.8-7.0)	.13	2.6 (0.8-8.5)	.12	1.7 (0.5-6.1)	.39
Cryptogenic	1 [Reference]	NA	1 [Reference]	NA	1 [Reference]	NA	1 [Reference]	NA
National Institute of Health Stroke Severity score at admission								
>3 (major stroke)	0.8 (0.5-1.2)	.23	NA	NA	1.2 (0.7-1.9)	.56	NA	NA
≤3 (minor stroke)	1 [Reference]	NA	NA	NA	1 [Reference]	NA	NA	NA
Cardiovascular health factors								
Hypertension	1.8 (1.1-2.7)	.01	1.9 (1.2-2.9)	.006	1.8 (1.1-2.9)	.02	1.5 (0.9-2.6)	.11
Dyslipidemia	0.9 (0.6-1.4)	.66	NA	NA	0.9 (0.5-1.5)	.68	NA	NA
Diabetes	1.4 (0.8-2.7)	.28	NA	NA	3.1 (1.8-5.3)	<.001	2.9 (1.7-4.9)	<.001
Current smoking	1.3 (0.7-2.2)	.40	NA	NA	1.8 (0.9-3.4)	.09	1.4 (0.7-2.8)	.33
Previous smoking	1.1 (0.7-1.8)	.78	NA	NA	1.8 (1.0-3.3)	.05	1.7 (0.9-3.2)	.09
Never smoked	1 [Reference]	NA	NA	NA	1 [Reference]	NA	1 [Reference]	NA
Excess alcohol use	0.8 (0.3-2.1)	.70	NA	NA	2.2 (1.1-4.3)	.03	2.0 (1.0-3.8)	.04
Illicit drug use^c^	1.1 (0.5-2.4)	.80	NA	NA	1.2 (0.5-2.8)	.73	NA	NA
Obesity	1.2 (0.7-2.0)	.44	NA	NA	0.7 (0.4-1.4)	.32	NA	NA
No. of vascular health factors								
≥3	1.0 (0.5-1.8)	.89	NA	NA	3.4 (0.8-14.2)	.21	NA	NA
1-2	0.6 (0.3-1.2)	.14	NA	NA	2.5 (0.6-10.6)	.10	NA	NA
0	1 [Reference]	NA	NA	NA	1 [Reference]	NA	NA	NA

Abbreviations: HR, hazard ratio; NA, not applicable.

^a^ HRs were calculated through age- and sex-adjusted Fine-Gray proportional hazard models.

^b^ HRs were calculated through multivariable Fine-Gray proportional hazard models and included factors with a *P* value < .10 in the age- and sex-adjusted analyses.

^c^ Information about illicit drug use was not available for 226 patients and were excluded from age- and sex-adjusted analysis.

## Discussion

In this cohort study, we found that the 5-year risk of any recurrent vascular event was 12.2% and was highest for patients with recurrent ischemic stroke and TIA rather than other vascular events. Patients with stroke due to CeAD had the highest short-term risk, and they remained at similar risk after 6 months. Factors associated with a short-term recurrence were CeAD, rare causes of stroke, and atherothrombotic stroke. The long-term risk was highest for patients with atherothrombotic stroke, while patients with cryptogenic stroke had the lowest risk after 5 years; long-term risk was also associated with cardioembolic stroke. Cardiovascular factors that were associated with the risk of any recurrent event after 5 years included the presence of hypertension, diabetes, and alcohol abuse at baseline.

We reported an incidence of recurrent vascular events that was comparable with previous studies that reported a 5-year risk of approximately 11.5%.^8,9,11^ We had anticipated a lower long-term risk compared with previous studies because our study was conducted in an era with much more rigorous secondary prevention. A possible explanation is that we included neuroimaging-proven index strokes. The risk of including stroke mimics as index events in previous studies^13,21^ of young adults may have led to an underestimation of the true recurrence risk because misdiagnosis of ischemic stroke is estimated to vary between 5% and 31% and is more common in young adults. Another reason could be that we included TIAs as an outcome event, which are often based on clinical diagnosis, resulting in a possible overestimation of the risk due to misclassification.^20^ Studies that did not include TIAs as an outcome reported a slightly higher risk of 11.5% to 15.0%,^8,9^ compared with the 9.5% that we found in our sensitivity analysis. An Italian multicenter cohort study^11^ that also included TIA as an outcome reported a 5-year risk of 11.5%, which is comparable to the 12.2% in our study.

The risk of recurrent vascular events in young patients after a cryptogenic stroke was found to be low: 3% in the first 6 months with annual decreases, which is in line with previous studies.^8,9^ This finding raises the question of whether these patients should be treated with long-term (often life-long) antithrombotic therapy; this is an important issue because recent studies^22^ have reported a risk of bleeding that is almost equal to the risk of ischemic events in young patients. Thus, the benefits of antithrombotic treatment may not always outweigh the risk of bleeding in young patients with such a low risk of recurrent ischemia. The incidence rate per 100 person-years between 6 months and 1 year for patients with cryptogenic stroke (0.5) was similar to the rate for patients with a CeAD (0.3). Although it is generally considered safe to discontinue antithrombotic treatment after 6 months for patients with a CeAD, the safety of discontinuing antithrombotic treatment after a cryptogenic stroke remains unclear. This clinically relevant dilemma warrants further attention, preferably in a randomized trial.

Our study indicates that young patients with a CeAD remain at high short-term risk of recurrence. Even though a CeAD shows a high cumulative risk after 5 years, the risk is highest in the first 6 months, and it is not associated with a long-term event. A possible explanation for this finding is that CeAD increases the early risk of intraluminal thrombi at the location of the tear, whereas intimal recovery recanalization of the vessel often occurs within 6 months.^23,24^ Therefore, fast recognition of CeAD-related clinical symptoms (such as trivial trauma, headaches, or neck pain shortly before the event) and imaging to demonstrate the intramural hematoma, followed by immediate antithrombotic treatment, is vital.^25^ Future studies should focus on determining optimal treatment, with regard to both antithrombotic treatment and endovascular therapy with stent placement in a selected group of patients who are at high-risk.^26^

When the TOAST classification^27^ was developed, certain stroke causes could not yet be identified and were classified cryptogenic, whereas other, rarer causes were classified into 1 category. For example, both CeAD and antiphospholipid syndrome are classified together in the rare cause category. However, antiphospholipid syndrome has been associated with a lifetime increased risk of venous and arterial thrombosis, whereas we have shown that patients with CeAD only have a short-term higher risk of recurrent stroke.^28,29,30^ Thus, 1 category of the TOAST classification contains multiple causes that are studied together but differ highly in terms of prognosis and appropriate secondary prevention strategies. This indicates that if we want to investigate the prognosis on a more individual level for young patients with ischemic stroke, the current cause classification system might not suffice, and we need to identify and study subtypes of causes in more detail.

## Limitations

There are some study limitations that need to be addressed. First, recurrent vascular events were self-reported by participants, which may be subject to recall-bias and may result in an overestimation or underestimation of the number of vascular events. However, we adjudicated all reported events and cross-validated this approach in patients who reported no events, showing a 100% agreement. Second, we excluded patients who died within 30 days of the index event, and the median NIHSS score at admission was low; this indicates that we included mostly patients with less severe stroke, and, thus, these results are not generalizable to severely affected young patients. A large Chinese study^31^ found that patient with mild NIHSS scores had a lower risk of recurrent vascular events compared with patients with severe or moderate NIHSS scores. Third, if diagnostic work-up was incomplete, the cause of the stroke was classified as cryptogenic, which could have led to misclassification, and, thus, an overestimation of cryptogenic strokes.

## Conclusions

In conclusion, our results suggest that despite the implementation of rigorous secondary prevention, 1 of 8 young patients experiences a recurrent vascular event within 5 years. Cause of stroke, including causes that remain concealed when grouped within the routinely used TOAST classification, might play an important role, and future research should implement subgroup analyses to investigate personalized secondary prevention strategies.

## Notes

Supplement 1. **eAppendix.** Modified TOAST Classification **eTable 1.** Diagnostic Work-Up in Cryptogenic Patients (n = 302) **eTable 2.** Distribution of Multiple Cases (n = 57) **eTable 3.** Cause of Stroke Based on the Modified TOAST Classification Stratified by Sex and Age **eTable 4.** Use of Antithrombotic Treatment at Discharge and Time of Recurrent Event

Supplement 2.
Data Sharing Statement
