# Supplementary material for: Short-Term and Long-Term Risk of Recurrent Vascular Event by Cause After Ischemic Stroke in Young Adults
Source: JAMA Netw Open. 2024 Feb 20;7(2):e240054. doi: 10.1001/jamanetworkopen.2024.0054 (PMC10879951; doi:10.1001/jamanetworkopen.2024.0054)
Supplement: Supplement 1. — eAppendix. Modified TOAST Classification eTable 1. Diagnostic Work-Up in Cryptogenic Patients (n = 302) eTable 2. Distribution of Multiple Cases (n = 57) eTable 3. Cause of Stroke Based on the Modified TOAST Classification Stratified by Sex and Age eTable 4. Use of Antithrombotic Treatment at Discharge and Time of Recurrent Event [file jamanetwopen-e240054-s001.pdf]

Supplemental Online Content

Verburgt E, Hilkens NA, Ekker MS, et al. Short-term and long-term risk of recurrent vascular event by cause after ischemic stroke in young adults. *JAMA Netw Open*. 2024;7(2):e240054. doi:10.1001/jamanetworkopen.2024.0054

eAppendix: Modified TOAST Classification

eTable 1. Diagnostic Work-Up in Cryptogenic Patients (n=302)

eTable 2. Distribution of Multiple Causes (n=57)

eTable 3. Cause of Stroke Based on the Modified TOAST Classification Stratified by Sex and Age

eTable 4. Use of Antithrombotic Treatment at Discharge and Time of Recurrent Event

This supplemental material has been provided by the authors to give readers additional information about their work.

© 2024 Verburgt E et al. *JAMA Network Open*.

eAppendix. Modified TOAST Classification

1a. Atherothrombotic: Stroke patients with an ipsilateral internal carotid stenosis ≥50%, or an ipsilateral stenosis ≥50% of another cervical or intracranial artery, or mobile thrombus in the aortic arch.

1b. Likely atherothrombotic: Stroke patients with no evidence of atherothrombotic stroke as defined in criteria 1, with an ipsilateral internal carotid stenosis <50%, or an ipsilateral stenosis <50% of another cervical or intracranial artery, or aortic arch plaques ≥4 mm in thickness without a mobile component, or a history of myocardial infarction or coronary revascularization, or a history of documented peripheral arterial disease, or at least two risk factors for atherosclerotic disease: arterial hypertension (treated or known blood pressure before stroke ≥140/90mm Hg or hypertensive retinopathy), diabetes mellitus (treated, or known blood fasting glucose ≥7 mmol/L), current smoking (or stopped smoking within the last 6 months) or high cholesterol (treated or known low-density lipoprotein before the stroke ≥160 mg/dl or 4.1 mmol/L).

2. Small vessel disease: Stroke patients with a small deep infarct measuring <15 mm on MRI (or CT) in the territory corresponding to symptoms, in a patient presenting a clinical syndrome compatible with a small deep infarct.

3. Cardioembolic: Stroke patients with at least one cardiac source for an embolus.

4a. Rare cause: Stroke patients who had another etiology.

4b. Cervical artery dissection: Stroke patients who had a carotid- or vertebral artery dissection.

5. Cryptogenic: Stroke patients who did not meet criteria for the groups defined above, maybe with incomplete evaluation.

6. Multiple: Patients with two or more etiologies defined in 1-4.

© 2024 Verburgt E et al. *JAMA Network Open*.

eTable 1. Diagnostic Work-Up in Cryptogenic Patients (n=302)

	Number of patients (%)
Complete work-up[a]	232 (76.8)
Imaging of the carotid arteries[b]	291 (96.4)
Screening antiphospholipid antibodies[c]	274 (90.7)
Electrocardiogram[d]	299 (99.0)
Holter monitoring[e]	272 (90.1)
TTE or TEE[f]	282 (93.4)

Abbreviation: TTE, transthoracic echocardiography; TEE, transesophageal echocardiography.
[a] Complete work-up was defined as all of the following diagnostics performed: imaging of the carotid arteries, antiphospholipid antibodies, electrocardiogram, minimum of 24 hours Holter monitoring and any echocardiography.
[b] Imaging of the carotid arteries was done through computer tomography angiography, magnetic resonance angiography or duplex. From the 11 patients who did not receive imaging of the carotid arteries, eight had an infarct in the posterior territory.
[c] Antiphospholipid antibodies was determined by screening anticardiolipin antibody of immunoglobulin G, lupus anticoagulants or beta 2 glycoprotein.
[d] For two patients, it was not known if they had received electrocardiogram monitoring.
[e] Holter monitoring was defined as a minimum of 24-hour monitoring. For three patients, it was not known if they had received Holter monitoring. 21 of the patients without Holter monitoring were included in the study before trials of patent foramen ovale closure in 2017.
[f] For three patients, it was not known if they had received TTE or TEE. 13 patients without TTE or TEE were included in the study before trials of patent foramen ovale closure in 2017.

© 2024 Verburgt E et al. *JAMA Network Open*.

eTable 2. Distribution of Multiple Causes (n=57)

	Atherothrombotic	Likely atherothrombotic	Cardioembolic	Small vessel disease	Rare cause
Atherothrombotic					
Likely atherothrombotic	0				
Cardioembolic	2	12			
Small vessel disease	0	2	1		
Rare cause	6	18[a]	9	6	1[b]

Abbreviation: n, number

[a] One patient was diagnosed with three causes: three cardiovascular risk factors, cervical artery dissection and antiphospholipid syndrome.

[b] One patient was diagnosed with both cervical artery dissection and antiphospholipid syndrome.

© 2024 Verburgt E et al. *JAMA Network Open*.

eTable 3. Cause of Stroke Based on the Modified TOAST Classification Stratified by Sex and Age

	Total	Atherothrombotic	Likely atherothrombotic	Small vessel disease	Cardioembolic	Rare causes	Cervical artery dissection	Cryptogenic	Multiple causes
**Sex (n(%)),** p-value= <0.001									
Men	632 (100)	34 (5.4)	89 (14.1)	104 (16.5)	108 (17.1)	47 (7.4)	83 (13.1)	136 (21.5)	31 (4.9)
Women	584 (100)	18 (3.1)	77 (13.2)	61 (10.4)	92 (15.8)	83 (14.2)	61 (10.4)	166 (28.4)	26 (4.5)
**Age (n(%)),** p-value= <0.001									
18-29 years	124 (100)	1 (0.8)	4 (3.2)	4 (3.2)	41 (33.1)	19 (15.3)	17 (13.7)	36 (29.0)	2 (1.6)
30-34 years	92 (100)	1 (1.1)	8 (8.7)	8 (8.7)	17 (18.5)	19 (20.7)	12 (13.0)	24 (26.1)	3 (3.3)
35-39 years	154 (100)	1 (0.6)	15 (9.7)	16 (10.4)	25 (16.2)	21 (13.6)	21 (13.6)	48 (31.2)	7 (4.5)
40-44 years	296 (100)	9 (3.0)	42 (14.2)	45 (15.2)	44 (14.9)	31 (10.5)	31 (10.5)	79 (26.7)	15 (5.1)
45-49 years	550 (100)	40 (7.3)	97 (17.6)	92 (16.7)	73 (13.3)	40 (7.3)	63 (11.5)	115 (20.9)	30 (5.5)

© 2024 Verburgt E et al. *JAMA Network Open*.

eTable 4. Use of Antithrombotic Treatment at Discharge and Time of Recurrent Event

	N of patients	Missing	Single antiplatelet	Dual antiplatelet	Anticoagulation[a]	Other antithrombotic treatment	No antithrombotic treatment	Not started yet at time of recurrent event
**Discharge**								
Total (n(%))	1216	3 (0.2)	894 (73.5)	224 (18.4)	64 (5.3)	22 (1.8)[b]	9 (0.7)	NA
TOAST classification (n(%))								NA
Atherothrombotic	52	1 (1.9)	42 (80.8)	9 (17.3)	0 (0)	0 (0)	0 (0)	
Likely atherothrombotic	166	0 (0)	138 (83.1)	23 (13.9)	3 (1.8)	1 (0.6)	1 (0.6)	
Small vessel disease	165	0 (0)	130 (78.8)	35 (21.2)	0 (0)	0 (0)	0 (0)	
Cardioembolic	200	0 (0)	121 (60.5)	37 (18.5)	32 (16.0)	8 (4.0)	2 (1)	
Rare causes	130	1 (0.8)	94 (72.3)	12 (9.2)	12 (9.2)	6 (4.6)	5 (3.8)	
Cervical artery dissection	144	0 (0)	114 (79.2)	24 (16.7)	5 (3.5)	1 (0.7)	0 (0)	
Cryptogenic	302	1 (0.3)	218 (72.2)	74 (24.5)	7 (2.3)	1 (0.3)	1 (0.3)	
Multiple causes	57	0 (0)	37 (64.9)	10 (17.5)	5 (8.8)	5 (8.8)	0 (0)	
**Time of recurrent event**								
Total (n(%))	137	15 (10.9)	84 (68.9)	14 (11.5)	10 (8.2)	0 (0)	2 (1.6)	12 (9.8)
TOAST classification (n(%))[c]								
Atherothrombotic	11	0 (0)	10 (90.9)	1 (9.1)	0 (0)	0 (0)	0 (0)	0 (0)
Likely atherothrombotic	19	3 (15.8)	12 (63.2)	2 (10.5)	0 (0)	0 (0)	0 (0)	2 (10.5)
Small vessel disease	23	4 (17.4)	18 (78.3)	1 (4.3)	0 (0)	0 (0)	0 (0)	0 (0)
Cardioembolic	20	0 (0)	11 (55.0)	1 (5.0)	6 (30.0)	0 (0)	1 (5.0)	1 (5.0)
Rare causes	18	1 (5.6)	10 (55.6)	1 (5.6)	2 (11.1)	0 (0)	1 (5.6)	3 (16.7)
Cervical artery dissection	21	4 (19.0)	9 (42.9)	4 (19.0)	0 (0)	0 (0)	0 (0)	4 (19.0)
Cryptogenic	17	2 (11.8)	11 (64.7)	3 (17.6)	1 (5.9)	0 (0)	0 (0)	0 (0)
Multiple causes	8	1 (12.5)	3 (37.5)	1 (12.5)	1 (12.5)	0 (0)	0 (0)	2 (25.0)

[a]Among 64 participants using oral anticoagulants at discharge, 26 were using direct oral anticoagulants (DOAC) and 38 were using vitamin K antagonists. Among 10 participants with a recurrent event on oral anticoagulants, 5 were using a DOAC and 5 were using vitamin K antagonists.

[b]Other antithrombotic treatments were a combination of antiplatelets and anticoagulation (n=11), low molecular weight heparin (n=10) and unknown which type of antithrombotic treatment (n=1).

[c]The TOAST classification regards the cause of stroke of the index event.

© 2024 Verburgt E et al. *JAMA Network Open*.
