# Supplementary material for: Short-Term and Long-Term Risk of Recurrent Vascular Event by Cause After Ischemic Stroke in Young Adults
Source: JAMA Netw Open. 2024 Feb 20;7(2):e240054. doi: 10.1001/jamanetworkopen.2024.0054 (PMC10879951; doi:10.1001/jamanetworkopen.2024.0054)
Supplement: Supplement 2. — Data Sharing Statement [file jamanetwopen-e240054-s002.pdf]

Data Sharing Statement

Verburgt. Short-Term and Long-Term Risk of Recurrent Vascular Event by Cause After Ischemic Stroke in Young Adults. *JAMA Netw Open*. Published February 20, 2024. doi:10.1001/jamanetworkopen.2024.0054

Data
Data available: Yes
Data types: Other (please specify)
Additional Information: Data is available upon request. Requests should be sent to the corresponding author for further information.
How to access data: Data is available upon request. Requests should be sent to the corresponding author for further information.
When available: With publication

Supporting Documents
Document types: Other (please specify)
Additional Information: Data is available upon request. Requests should be sent to the corresponding author for further information.
How to access documents: Data is available upon request. Requests should be sent to the corresponding author for further information.
When available: With publication

Additional Information
Who can access the data: Data is available upon request. Requests should be sent to the corresponding author for further information.
Types of analyses: Data is available upon request. Requests should be sent to the corresponding author for further information.
Mechanisms of data availability: Data is available upon request. Requests should be sent to the corresponding author for further information.
Any additional restrictions: Data is available upon request. Requests should be sent to the corresponding author for further information.
